# Analysis of the Dielectric Properties of Alkali-Free Aluminoborosilicate Glasses by Considering the Contributions of Electronic and Ionic Polarizabilities in the GHz Frequency Range

**DOI:** 10.3390/ma17061404

**Published:** 2024-03-19

**Authors:** Linganna Kadathala, Young-Ouk Park, Myoung-Kyu Oh, Won-Taek Han, Bok Hyeon Kim

**Affiliations:** Advanced Photonics Research Institute, Gwangju Institute of Science and Technology, 123 Cheomdangwagi-ro, Buk-gu, Gwangju 61005, Republic of Korea; lingannagist@gist.ac.kr (L.K.); youngouk@gist.ac.kr (Y.-O.P.); omkyu@gist.ac.kr (M.-K.O.); wthan@gist.ac.kr (W.-T.H.)

**Keywords:** alkali-free aluminoborosilicate glasses, dielectric constant, dielectric polarizability, oxide ion polarizability, PCB application

## Abstract

Recently, the investigation of the dielectric properties of glasses in the GHz frequency range has attracted great interest for use in printed circuit boards (PCBs) as a reinforcing material in the application of high-speed 5G/6G communications. In particular, glasses with low dielectric properties are a prerequisite for high-frequency applications. In this study, the GHz dielectric properties of alkali-free aluminoborosilicate glasses without and with La_2_O_3_ were analyzed using the Clausius–Mossotti equation where both the electronic and ionic polarizabilities contribute to the dielectric constant. The dielectric polarizability (α_D_) and oxide ion polarizability (α_O2−_) were calculated from the measured dielectric constant (ε_GHz_) at 1 GHz and the glass density. The dielectric constants (ε_opt_) at the optical frequencies and electronic polarizabilities (α_e_) of the glasses were calculated from the refractive index measured at 633 nm and the glass density. The ε_GHz_ values were found to be significantly higher than the ε_opt_ values in both series of glasses, due to the ionic polarizability (α_i_), which contributes additionally to the ε_GHz_. The lower dielectric constants of the La_2_O_3_-incoporated glasses than that of the reference glass without La_2_O_3_ may be due to the lower ionic polarizability originated from the incorporation of the high cation field strength of the La^3+^ ions.

## 1. Introduction

In recent years, the needs to increase data transmission rates and network connectivity have become more urgent because of the rapid progression in the development of 5G/6G telecommunication, the internet of things (IoT), autonomous vehicles, low orbit satellite communication, etc. [1,2,3,4,5,6,7,8]. In order to realize these emerging technologies, millimeter (mm)-wave devices require improvements in their electronic designs [1,3,5,7,8]. In addition to the advanced electronic designs, satisfying the requirement of a low dielectric property is very important to enable fast signal transmission, as the signal loss is mostly determined by the dielectric properties of insulators and their interface with conductors in the mm-wave frequency range [1,3,5,7,8]. The low dielectric property shortens propagation delay and mitigates signal loss and also leverages larger device geometries that decrease sensitivity to fabrication tolerances. Investigations on the dielectric property are essential for understanding the glass structure as it relates to the polarizability of constituent ions forming the glass [9,10,11,12,13]. Moreover, they can provide insight into the chemical structure of the glass at GHz frequencies, where the electronic and ionic mechanisms contribute to the polarizability. Glass fibers with a lower dielectric constant (≤4.9) and dissipation factor (≤0.005) are important materials for reinforcing electronic products in the application of high-speed telecommunications [7,8,14].

In this connection, the characterization of the dielectric properties of glass materials in the GHz frequency range has gained growing interest in designing new glass compositions for use in printed circuit boards (PCBs) as a reinforcing material for high-speed data transmission applications. Since the dielectric constant (ε) is calculated based on the polarizability and density of glasses through the Clausius–Mossotti (CM) equation [9,10,11,12,13,14,15,16], the investigation of polarizability characteristics such as dielectric polarizability (α_D_), oxide ion polarizability (α_O2−_), and optical basicity (Λ) is important for understanding the dielectric responses of glasses in the GHz frequency region. The dielectric properties of glasses are also influenced by the low-frequency (GHz–THz) and the high-frequency (optical) modes [9,10,11,12,14,15,16]. The dielectric constant at a GHz frequency is a result of contributions from the electronic and ionic polarizabilities, whereas the dielectric constant at an optical frequency is a result of contribution from the electronic polarizability [7,14,15,16]. The CM equation is the method most extensively used to investigate the dielectric properties of materials. The concise formula reveals that the decisive factors of the dielectric constant are the molecular dielectric polarizability and molar volume. The molecular dielectric polarizability of a compound can be obtained through the summation of an individual cation and anion polarizabilities according to the additivity rule.

Oxide ion polarizability (α_O2−_) is considered to be an important parameter for reflecting the overall glass characteristics and is strongly influenced by its structure and chemical environment [17,18,19]. In addition, the α_O2−_ is one of the main factors contributing to the polarizability of oxide glasses [20,21,22,23,24]. α_O2−_ for single oxides [20,21,23], binary glass systems (silicate, borate, tellurite, and germanate) [19,21,23], and ternary glass systems (silicate, tellurite, and germanate) [23] has been estimated from the refractive index values measured at visible wavelength, yielding different values for each glass system. For instance, α_O2−_ varies from 1.540 to 1.627 Å^3^ in a Na_2_O-SiO_2_ glass system, as the Na_2_O content increases from 15 to 30 mol% [23]. Meanwhile, α_O2−_ varies from 2.293 to 1.914 Å^3^ in a Na_2_O-Nb_2_O_5_-SiO_2_ glass system, as the Na_2_O content decreases from 33.3 to 16.7 mol% and SiO_2_ content increases from 33.3 to 66.6 mol% [23]. Optical basicity (Λ) is a key parameter for characterizing the glass structure and ionicity properties, and it also reflects the oxide ion polarizability of the glass [17,18,19]. An increase in ionicity in a glass increases the Λ because oxygen moves closer to an anionic state (O^2−^), thereby acquiring the ability to transfer its electron cloud to the surrounding environment.

In view of the important considerations above, various investigations on the dielectric properties of glasses have been conducted in the GHz–THz frequency range using the well-known CM equation [14,15,16]. Lanagana et al. [14] investigated the permittivity and loss tangent values of alkali- and alkaline earth-modified silicate glasses at 10 GHz and estimated the α_D_ of the glasses from the permittivity and molar volume using the CM equation. Wada et al. [15] investigated the dielectric properties of oxyfluorosilicate glasses in the THz region using terahertz time-domain transmission spectroscopy (THz-TDS). It was shown that the dielectric properties of the glasses result from the contributions of both the electronic and ionic polarizabilities. Naftaly and Miles [16] conducted a study on the absorption coefficients and refractive indices of silicate glasses using THz-TDS and found that the complex permittivity in the 0.1–3 THz frequency range was significantly influenced by ionic polarization. They found that the addition of oxide modifiers to the silicate glass matrix led to a pronounced increase in polarizability. However, few studies have been conducted on the characterization of dielectric properties of alkali-free aluminoborosilicate glasses in the GHz frequency range.

Aluminoborosilicate glasses are attractive materials for composite structure applications, including PCBs, owing to their low dielectric property, low coefficient of thermal expansion, high thermal stability, high mechanical stability, etc. [25,26,27,28,29,30,31,32]. Recently, alkali-free aluminoborosilicate glass compositions incorporating rare earth (RE) species have attracted considerable attention for the development of low-dielectric glass fibers because of their promising ability to improve the viscosity and dielectric properties [30,31,32]. Recently, we fabricated alkali-free aluminoborosilicate glasses by varying SiO_2_ and La_2_O_3_ contents and investigated the structural, thermal, and MHz–GHz dielectric properties of the glasses.

In this study, through this analysis, we aimed to understand the polarizability characteristics of the glasses that influence their dielectric properties at GHz and optical frequencies. The dielectric and polarizability properties at 1 GHz were investigated in two series of alkali-free aluminoborosilicate glasses fabricated by varying the SiO_2_ and La_2_O_3_ contents. The α_D_, α_O2−_, and Λ values of the glasses were obtained from the measured ε_GHz_ and glass density properties using the CM equation. The refractive indices of the glasses were measured at 633 nm, and the dielectric constants (ε_opt_) at the optical frequencies and electronic polarizabilities (α_e_) of the glasses were calculated from the measured refractive indices. For a comparative study, we also examined the difference between the GHz and optical dielectric constants, from which the electronic and ionic polarizabilities were determined. Furthermore, the relationships between the dielectric and polarizability properties at 1 GHz and at 633 nm and the physical characteristics of the glasses with respect to the glass compositions were examined.

## 2. Materials and Methods

### 2.1. Fabrication of Alkali-Free Aluminoborosilicate Glasses

Two series of alkali-free aluminoborosilicate glasses were prepared using the melt-quenching method. The chemical compositions of two series of alkali-free aluminoborosilicate glasses without and with La_2_O_3_ (referred to as GDMC-Si and GDMC-La, respectively) are listed in Table 1. The GDMC-Si glasses were fabricated by varying the SiO_2_ and Al_2_O_3_ contents, and the rest of the chemical components (B_2_O_3_, CaO, and MgO) were kept at nearly constant in the glasses. The GDMC-La glass compositions were designed from the reference GDMC-Si59 glass and fabricated by substituting La_2_O_3_ for (Al_2_O_3_ + MgO). High-purity powders of SiO_2_, Al_2_O_3_, B_2_O_3_, MgCO_3_, CaCO_3_, and La_2_O_3_ (Kojundo, Sakado, Japan, ≥99.9%) were weighed using a balance and mixed using a ball mill for 2 h. A homogeneous mixture of each batch composition was melted at 1650 °C in an electric furnace for the duration of 3 h. The viscous glass melt was then quenched by pouring it onto a pre-heated brass plate. The glasses were annealed at 650 °C for 2 h to release residual stress in the glass and naturally cooled down to room temperature. The glass samples were cut and polished to the desired dimensions for the optical and dielectric measurements. The glasses with a La_2_O_3_ concentration up to 1.2 mol% showed the characteristics of vitreous phases since they were transparent and homogeneous. Beyond this concentration, the crystallization phases appeared in the melt, which characterize the solubility limit of La_2_O_3_ for this composition.

### 2.2. Characterization of Alkali-Free Aluminoborosilicate Glasses

The densities of the glasses were measured using a densimeter (Alfa Mirage, MD-300S, Seongnam-si, Republic of Korea). The density of each glass sample was measured at multiple times, and the deviation of the measured density was approximately ±0.003 g/cm^3^.

Molar volume (V_m_) was determined from the glass density (ρ) and molecular weight (M_i_) of each glass component weighted by its molar fraction (x_i_):

(1) $$V_{m}=\frac{\sum_{i}x_{i}M_{i}}{\rho }$$

Oxygen packing density (ρ_ox_) was determined using the following equation:

(2) $$\rho_{ox}=\frac{1}{V_{m}}\sum_{i}x_{i}n_{i}$$

where n_i_ represents the number of oxygen atoms in each glass component, i.e., 2 for SiO_2_, 3 for Al_2_O_3_, 3 for B_2_O_3_, 1 for MgO, 1 for CaO, and 3 for La_2_O_3_. To some extent, the structure of the glass can be viewed as oxygen atoms densely packed with other elements. The molar volume of oxygen (V_ox_) indicates the degree of densely packed oxygen and can be calculated from V_m_ and n_i_ using the following equation:

(3) $$V_{\mathrm{ox}}=V_{m}\left(\frac{1}{\sum_{i}x_{i}n_{i}}\right)$$

The dielectric properties of the glasses were measured at 1 GHz using the parallel plate method with a sensor probe (Keysight, 16453A, Santa Rosa, CA, USA) and an RF impedance analyzer (Agilent, E4991A, Santa Clara, CA, USA) [33]. Optical absorption spectra of glasses were measured using a UV-VIS-NIR spectrometer (Perkin Elmer, LAMBDA 950, Westford, MA, USA) in the wavelength region between 300 and 2500 nm with a spectral resolution of 5 nm. The refractive indices of the glasses were measured using a prism coupler apparatus (SAIRON, SPA4000, Gwangju, Republic of Korea) at various wavelengths of 633, 830, 1310, and 1550 nm. Polished thin glass sheets with a size of 85 × 85 × 0.78 mm^3^ were used for the measurement.

## 3. Results

### 3.1. Physical Properties

Density is a macroscopic parameter that reflects the polymerization of the glass network structure and depends on the glass composition and coordination number of the atoms [30,34,35,36]. Table 2 lists the physical properties of the GDMC-Si and GDMC-La glass systems.

**Figure 1:**
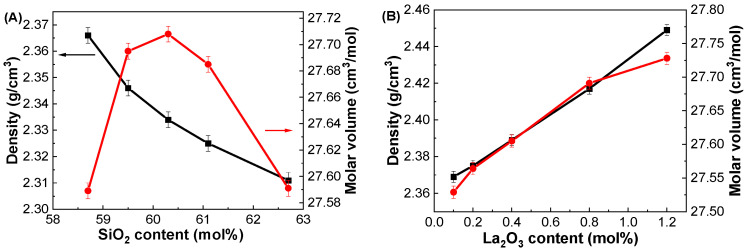
Variations in density (black squares) and molar volume (red circles) as functions of (**A**) SiO_2_ and (**B**) La_2_O_3_ contents in the GDMC-Si and GDMC-La glasses.

Figure 1 shows the variations in ρ and V_m_ as functions of SiO_2_ content in the GDMC-Si glasses. As shown in Figure 1A, the ρ continuously decreased from 2.366 to 2.311 g/cm^3^ as the SiO_2_ content increased from 58.7 to 62.7 mol% in the glasses. Meanwhile, the V_m_ initially increased and subsequently decreased with increasing SiO_2_ content. The decrease in ρ of the glasses with increasing SiO_2_ content is attributed to the progressive substitution of SiO_2_, which has a lighter molar mass of 60.08 g/mol compared to the 101.96 g/mol of Al_2_O_3_. In addition, the observed trends of ρ and V_m_ in the glasses with the substitution of SiO_2_ for Al_2_O_3_ are attributed to the high-field strength modifier cations (R^2+^: Mg^2+^, Ca^2+^) [30,37,38]. In the alkali-free aluminoborosilicate glasses, the R^2+^ ions prefer to form NBOs rather than to compensate the negatively charged [BO_4_]^−^ and [AlO_4_]^−^ tetrahedral units when the RO/Al_2_O_3_ ratio is greater than 1 [30,37,38]. Therefore, the glass network is depolymerized in the GDMC-Si glasses with the substitution of SiO_2_ for Al_2_O_3_ because of the increase in NBOs, thus resulting in a decrease in ρ and an increase in V_m_ in the glasses. Interestingly, an anomalous molar volume decrease was observed with increasing the SiO_2_ content in the range from 60.3 to 62.7 mol% in the glasses.

Figure 1B shows the variations in ρ and V_m_ as functions of La_2_O_3_ content in the GDMC-La glasses. The density change was more pronounced in the glasses with the incorporation of La_2_O_3_. The ρ linearly increased in the range of 2.369–2.449 g/cm^3^ with a small substitution of La_2_O_3_ content for (Al_2_O_3_ + MgO) in the range of 0.1 to 1.2 mol%. Meanwhile, the V_m_ increased in the range of 27.529–27.728 cm^3^/mol with the corresponding incorporation of La_2_O_3_ content. The increase in the ρ of the glasses is attributed to the progressive incorporation of the heavier molar mass component of La_2_O_3_ (325.81 g/mol) substituted for the relatively lighter molar mass components of Al_2_O_3_ (101.96 g/mol) and MgO (40.30 g/mol). In addition, the incorporated La_2_O_3_ provides free oxygens (O^2−^) and transforms some of the [BO_3_] trigonal pyramid units into the [BO_4_] tetragonal bipyramid units [31,35], thereby increasing the polymerization of the glass network and in turn leading to an increase in ρ. Moreover, the La_2_O_3_ species possesses a high cation field strength that can attract the NBOs and complement the glass network structure, resulting in an increase in ρ. The increase in V_m_ in the GDMC-La glasses with increasing La_2_O_3_ content may be attributed to the larger ionic radius of La^3+^ ions (1.22 Å) compared to that of the other glass constituents. Similar trends in density and molar volume with the addition of La_2_O_3_ content were observed in the La_2_O_3_–Al_2_O_3_–SiO_2_ glass system [39]. Notably, the density values of the GDMC-La glasses were lower than those of the RE-doped aluminoborosilicate glasses containing RE oxides [32,35,40], ensuring their suitability for PCB applications.

**Figure 2:**
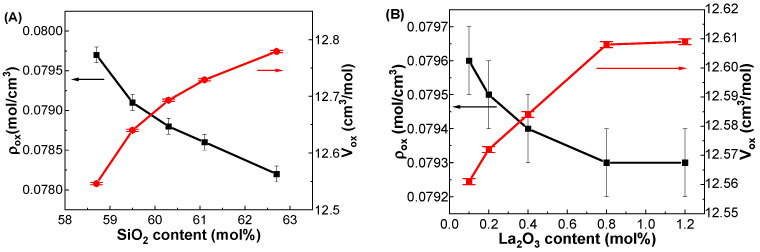
Variations in ρ_ox_ (black squares) and V_ox_ (red circles) with respect to the SiO_2_ and La_2_O_3_ contents in the (**A**) GDMC-Si and (**B**) GDMC-La glasses.

Figure 2A shows the variations in ρ_ox_ and V_ox_ as functions of SiO_2_ content in the GDMC-Si glasses. The ρ_ox_ continuously decreased from 0.0797 to 0.0782 mol/cm^3^, and the V_ox_ increased from 12.55 to 12.78 cm^3^/mol as the SiO_2_ content increases from 58.7 to 62.7 mol%, respectively. The decrease in ρ_ox_ and increase in V_ox_ in the glasses with increasing SiO_2_ content substituted for A_2_O_3_ may be due to the increase in tetrahedral units with fewer cross-links. This clearly indicates that the glass network structure is depolymerized with increasing the substitution of SiO_2_ for Al_2_O_3_ in the glasses. Figure 2B shows the variations in ρ_ox_ and V_ox_ as functions of La_2_O_3_ content in the GDMC-La glasses. The ρ_ox_ decreased from 0.0796 to 0.0793 mol/cm^3^, while the V_ox_ increased from 12.56 to 12.61 cm^3^/mol with increasing the substitution of La_2_O_3_ for (Al_2_O_3_ + MgO) in the range from 0.1 to 1.2 mol%. The trend observed in ρ_ox_ with the La_2_O_3_ content may be attributed to the high cation field strength of La^3+^ ions, which will improve the connectivity of the glass network by reducing the NBOs [31,32,35,40]. The increase in V_ox_ with increasing the La_2_O_3_ content in the glasses is attributed to the higher number of bonds formed, as La_2_O_3_ species provide free oxygens to polymerize the glass network structure. It is worthy to note that the increase in ρ_ox_ and decrease in V_ox_ with increasing the substitution of La_2_O_3_ in the GDMC-La glasses indicate the polymerization of glass network when compared with those in the reference GDMC-Si59 glass.

### 3.2. Dielectric and Polarizability Properties at GHz Frequencies

**Figure 3:**
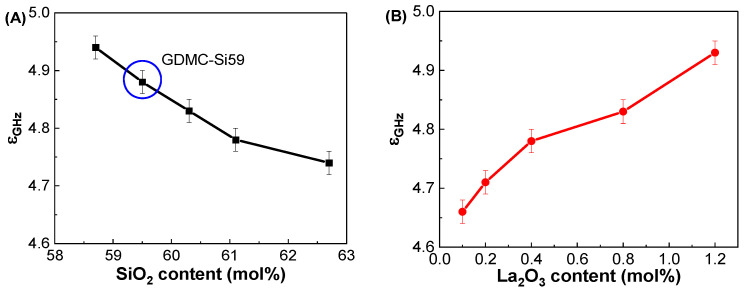
Variations in dielectric constant (ε_GHz_) at 1 GHz as functions of the SiO_2_ and La_2_O_3_ contents in the (**A**) GDMC-Si and (**B**) GDMC-La glass systems.

Figure 3A shows the ε_GHz_ values of the GDMC-Si glass system with respect to the SiO_2_ contents. The ε_GHz_ decreased from 4.94 to 4.74 with increasing the substitution of SiO_2_ for Al_2_O_3_ in the glasses. In the GDMC-La glasses, the ε_GHz_ increased from 4.66 to 4.93 with increasing the substitution of La_2_O_3_ for (Al_2_O_3_ + MgO), as shown in Figure 3B. The ε_GHz_ of the glasses mainly results from the polarizability contribution of their constituents [9,10,11,12,13,14,15,16]. Interestingly, with the incorporation of La_2_O_3_ up to 0.8 mol%, ε_GHz_ decreased by approximately 1 to 3% compared with that of the reference GDMC-Si59 glass. This decrease can be explained by the polymerization of the glass network. The addition of La_2_O_3_ to the glass matrix reduces the polarizable NBOs in the [SiO_4_] network of the GDMC-La glasses, leading to a decrease in the ε_GHz_ of the glasses. However, ε_GHz_ increased with a further increase in La_2_O_3_ content (1.2 mol%) compared with that of the reference glass, which might be due to the increase in free oxygens (O^2−^) through the relatively large La_2_O_3_ incorporation, and this would depolymerize the glass network reversely [31,32,35,40]. These results indicate that an increasing glass network connectivity could reduce α_D_ and in turn lead to a decrease in ε_GHz_ by introducing RE elements in the alkali-free aluminoborosilicate glasses up to certain levels. Table 3 summarizes the ε_GHz_ values measured at 1 GHz and the other polarizability properties of the GDMC-Si and GDMC-La glasses.

The dielectric properties of aluminoborosilicate glasses incorporated with RE ions were studied at MHz frequencies by Y. Yue et al. [30,31,35,40]. They found that the single- and co-doping of RE species can reduce the dielectric constant of the glasses because of the increase in the polymerization of the glass network structure. Several researchers have studied the dielectric responses and polarization mechanisms of alkali and alkaline earth-modified silicate glasses in the GHz–THz frequency range [7,9,10,11,12,14]. It was shown that the alkali-modified silicate glasses exhibit strong dielectric dispersion and show higher dielectric constants than the alkali-free glasses, which is due to the vibrations and migrations of alkali ions that are easily moved in the glass subjected to electromagnetic waves. The studies suggested that glasses with a low amount of alkali metal ions and less polarizable ions are required when they are applied as a high-frequency dielectric material.

According to the CM equation, α_D_ has relationships with ε_GHz_ and V_m_, and is expressed by the following equation [9,10,11,12,13,14,15,16]:

(4) $$\alpha_{D}=\left(\frac{3}{{4\pi \pi }_{A}}\right)\left[\left(V_{m}\right)\frac{\left(\varepsilon_{\mathrm{GHz}}-1\right)}{\left(\varepsilon_{\mathrm{GHz}}+2\right)}\right]$$

where V_m_ is the molar volume, and N_A_ is the Avogadro number (6.023 × 10^23^ mol^−1^). The α_D_ values of the alkali-free aluminoborosilicate glasses were calculated from the measured ε_GHz_ and ρ using Equations (1) and (4).

**Figure 4:**
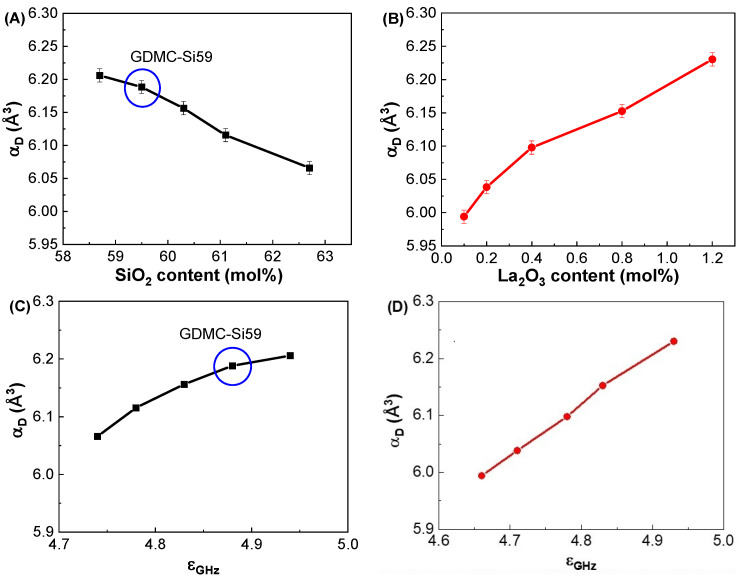
Variations in dielectric polarizabilities (α_D_) with respect to the SiO_2_ and La_2_O_3_ contents in the (**A**) GDMC-Si and (**B**) GDMC-La glasses; the relationships between the dielectric polarizability (α_D_) and dielectric constant (ε_GHz_) in the (**C**) GDMC-Si and (**D**) GDMC-La glass systems.

Figure 4A shows the variations in α_D_ with respect to the SiO_2_ contents in the GDMC-Si glasses. The α_D_ decreased from 6.21 to 6.07 Å^3^ with increasing the substitution of SiO_2_ for Al_2_O_3_ in the range of 58.7 to 62.7 mol%. The substitution of SiO_2_ for Al_2_O_3_ in the GDMC-Si glasses replaces the ionic [AlO_4_]^−^ tetrahedra with the covalent [SiO_4_] tetrahedra [41]. This decrease in the ionic bonding characteristics of the glass structure could result in the decrease in α_D_ in the glasses. Figure 4B shows the variations in α_D_ with respect to the La_2_O_3_ contents in the GDMC-La glasses. The α_D_ increased from 5.99 to 6.23 Å^3^ with increasing the substitution of La_2_O_3_ for (Al_2_O_3_ + MgO) in the range of 0.1 to 1.2 mol%. The α_D_ increased with increasing the substitution of La_2_O_3_ for (Al_2_O_3_ + MgO), which is attributed to the relative increase in polarizable NBOs caused by the incorporation of high cation field strength La^3+^ ions [30,31,32]. Interestingly, the α_D_ of the glasses with the La_2_O_3_ content up to 0.8 mol%, was lower than that of the reference glass (GDMC-Si59). As mentioned before, this result may be attributed to the fact that the La_2_O_3_ species can attract NBOs, and this would improve the connectivity of the glass network by transforming the boron and aluminum structural units, and the increase in the connectivity leads to a decrease in α_D_ [30,31,32]. Figure 4C,D show the variations in α_D_ with respect to ε_GHz_ in the GDMC-Si and GDMC-La glass systems, respectively. As shown in the figures, almost linear relationships between ε_GHz_ and α_D_ were found in both the glass systems.

The α_O2−_ was estimated from the α_D_ and cation polarizability (α_C_) according to the following equations [13,14]:

(5) $$\alpha_{O2-}=\frac{\alpha_{D}-\alpha_{c}}{X}$$

(6) $$\alpha_{C}=\sum_{i}x_{i}\alpha_{i}^{c}$$

(7) $$X=\sum_{i}x_{i}y_{i}$$

where x_i_ is the mole fraction of each component, y_i_ is the number of oxide ions, and $\alpha_{i}^{C}$ is the cation polarizability of each component. The known values were used for the cation ion polarizabilities (in Å^3^): Si^4+^, 0.87; Al^3+^, 0.79; B^3+^, 0.05; Ca^2+^, 3.16; Mg^2+^, 1.32; and La^3+^, 6.07 [13]. The calculated α_O2−_ values for the different glass compositions are summarized in Table 3.

**Figure 5:**
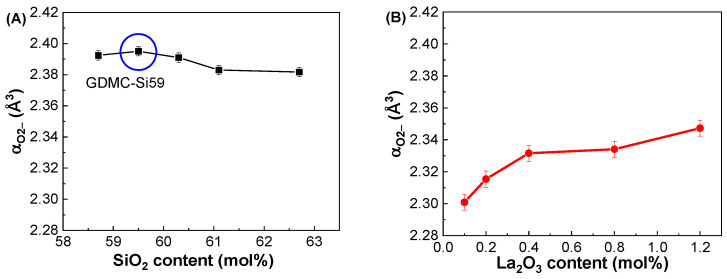

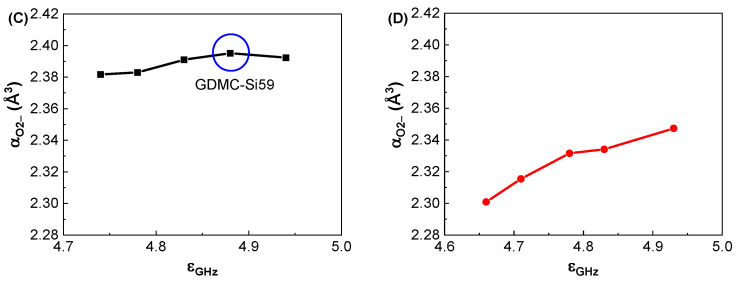
Variations in oxide ion polarizability (α_O2−_) with respect to the SiO_2_ and La_2_O_3_ contents in the (**A**) GDMC-Si and (**B**) GDMC-La glasses; the relationship between the oxide ion polarizability (α_O2−_) and dielectric constant (ε_GHz_) in the (**C**) GDMC-Si and (**D**) GDMC-La glasses.

Figure 5A depicts the compositional dependence of α_O2−_ in the GDMC-Si glasses. The α_O2−_ of the glasses was found to be nearly constant regardless of the SiO_2_ content substituted for Al_2_O_3_. The average α_O2−_ for the glasses was estimated to be 2.389 Å^3^. Figure 5B depicts the compositional dependence of α_O2−_ in the GDMC-La glasses. The α_O2−_ of the glasses slightly increased from 2.301 to 2.347 Å^3^ with the increase in La_2_O_3_ being from 0.1 to 1.2 mol%. This increase is caused by the La_2_O_3_ oxide that acts as a modifier similar to alkali oxides and increases the number of NBOs in aluminosilicate glasses [39]. Thus, La_2_O_3_ is regarded as a strong basic oxide, resulting in large α_O2−_ values in La_2_O_3_-doped silicate glasses. The average α_O2−_ value for the glasses was estimated to be 2.326 Å^3^. Notably, the magnitude of the α_O2−_ in the GDMC-La glasses was lower than that of the GDMC-Si glasses. The relatively smaller α_O2−_ values in the GDMC-La glasses may be originated from the high-field strength characteristic of the La_2_O_3_ species, which can improve the network connectivity by reducing the NBOs [30,31,32]. Figure 5C,D show the relationships between α_O2−_ and ε_GHz_ for the GDMC-Si and GDMC-La glass systems, indicating that the ε_GHz_ of alkali-free aluminoborosilicate glasses is affected by the polarizability properties of oxygen ions.

The Λ was also estimated from α_O2−_ using the following equation [17,18,19,20,21,22,23,24]:

(8) $$\Lambda =1.67\left(1-\frac{1}{\alpha_{O2-}}\right)$$

The calculated Λ values for the alkali-free aluminoborosilicate systems are summarized in Table 3.

**Figure 6:**
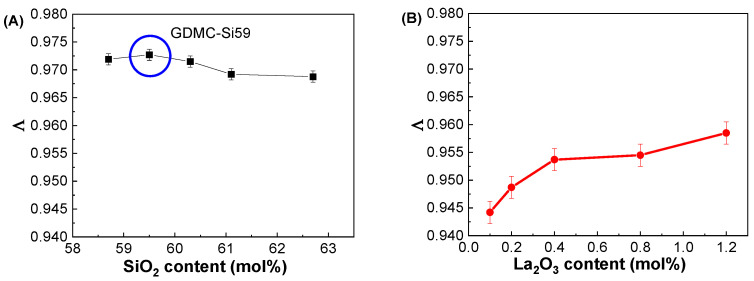

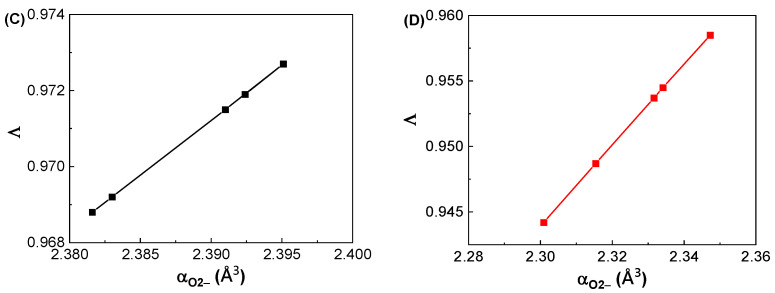
Variations in optical basicity (Λ) with respect to the SiO_2_ and La_2_O_3_ contents in the (**A**) GDMC-Si and (**B**) GDMC-La glasses; the relationship between the optical basicity (Λ) and oxide ion polarizability (α_O2−_) values in the (**C**) GDMC-Si and (**D**) GDMC-La glasses.

Figure 6A depicts the variations in Λ with respect to the SiO_2_ contents in the GDMC-Si glasses. A very small linear increase in Λ was observed with the substitution of SiO_2_ for Al_2_O_3_ in the glasses, and the averaged Λ value was 0.971. Figure 6B depicts the variations in Λ with respect to the La_2_O_3_ contents in the GDMC-La glasses. A slight increase in Λ from 0.944 to 0.959 was observed in the glasses with the increase in La_2_O_3_ substituted for (Al_2_O_3_ + MgO). The average Λ value of the glasses was 0.952. The GDMC-La glasses with La_2_O_3_ incorporation exhibit lower Λ values than the GDMC-Si glasses, and this indicates that the glasses have smaller polarizabilities and stronger chemical bond strengths compared to those of the GDMC-Si glasses [22,23,24]. Komatsu et al. [22] investigated Λ values for different binary and ternary silicate glasses from α_O2−_ values derived using the Lorentz–Lorenz equation. They found that glass compositions with large Λ values exhibit high electronic polarizability and weak chemical bond strengths. In addition, Komatsu and Dimitrov [23] investigated the α_O2−_ characteristics of binary and ternary tellurite glasses and found that high electronic polarizability and weak bond strengths could lead to large Λ values in the glasses. Figure 6C,D show the relationships between α_O2−_ and Λ in the GDMC-Si and GDMC-La glasses. The linear relationships between α_O2−_ and Λ were observed in both the glass systems, and these characteristics are consistent with those of the silicate glasses reported in the literature [20,21,22].

### 3.3. Dielectric and Polarizability Properties at Optical Frequencies

**Figure 7:**
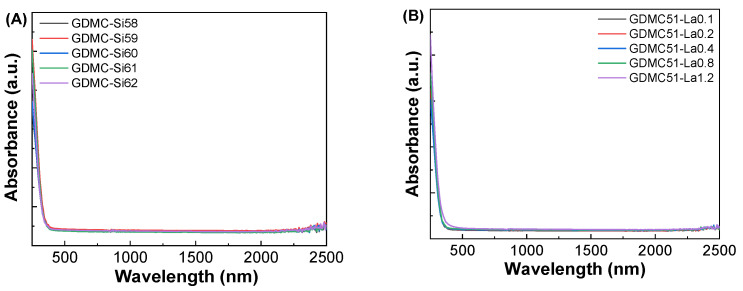

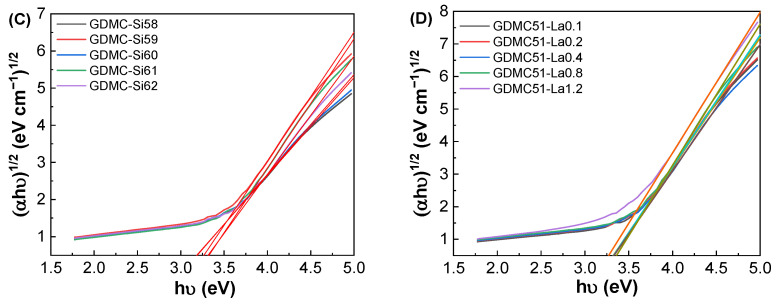
(**A**,**B**) Absorption spectra and (**C**,**D**) Tauc’s plots for the GDMC-Si and GDMC-La glasses.

Figure 7A,B show the UV-VIS-NIR absorption spectra of the GDMC-Si and GDMC-La glasses. As shown in the figure, the absorption spectra of two series of glasses show an exponential increase in the UV edge regions (~400 nm), indicating the amorphous nature of the glasses. Mott and Davis proposed a relationship that is used for the determination of optical bandgap energy (E_g_) in amorphous materials [42]:

(9) $$\alpha h\nu =B{\left(h\nu -E_{g}\right)}^{2}$$

where α is a linear absorption coefficient, B is a constant, and hν is the photon energy. The α is given by

(10) $$\alpha \left(\nu \right)=\frac{2.303\times A}{d}$$

where A refers to the optical absorbance, and d refers to the thickness of the glass sample.

Figure 7C,D show Tauc’s plots between (αhν)^1/2^ and hν for the GDMC-Si and GDMC-La glasses. The E_g_ values were determined by extrapolating the linear region of the curve to the hν axis, where αhν = 0. The obtained E_g_ values were found to be in the ranges of 3.17–3.32 eV and 3.20–3.35 eV for the GDMC-Si and GDMC-La glasses, respectively. It is noted that the overall trend of the E_g_ values increased with increasing the substitutions of SiO_2_ for Al_2_O_3_ and La_2_O_3_ for (Al_2_O_3_ + MgO) in both the glass systems. This indicates that the UV absorption edge was shifted to lower wavelengths with the increases in SiO_2_ and La_2_O_3_ contents in the studied glasses.

**Figure 8:**
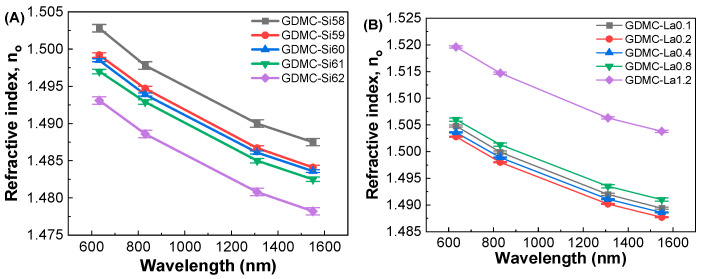
Wavelength dependence of the refractive indices in (**A**) GDMC-Si and (**B**) GDMC-La glass systems.

Figure 8 shows the refractive indices of the (A) GDMC-Si and (B) GDMC-La glass systems in the optical region at different wavelengths of 633, 830, 1310, and 1550 nm. As expected, the refractive index (n_o_) decreased with respect to the wavelength in both series of glasses. The refractive index was found to decrease from 1.5028 to 1.4931@633 nm with the substitution of SiO_2_ for Al_2_O_3_ in the GDMC-Si glasses. The decrease in n_o_ is ascribed to the lower polarizability of the glasses with the substitution of SiO_2_ for Al_2_O_3_. In the GDMC-La glasses, the refractive index increased from 1.5028 to 1.5196@633 nm upon La_2_O_3_ substitution at concentrations greater than 0.2 mol%. The increase in n_o_ is ascribed to the higher polarizability caused by the La_2_O_3_ species, which provide sufficient oxygen ions leading to the creation of NBOs in the glass structure. Among the studied glasses, the GDMC-La glasses showed higher refractive indices than those of the GDMC-Si glasses. The decrease or increase matched with the density trends for the same compositional changes in both series of glasses. A decrease in density can be expected to cause a decrease in the average electron density of the glasses. This reduced electron density will subsequently affect the refractive index. Notably, the refractive index values of the present glasses are slightly higher than those of the commercial fused silica (n_0_ = 1.458@589 nm) and Pyrex (n_0_ = 1.474@589 nm) [16]. Table 4 presents the refractive indices (n_o_), optical dielectric constants (ε_opt_), and electronic polarizabilities (α_e_) of the GDMC-Si and GDMC-La glass systems.

In order to compare the dielectric properties of the studied glasses in the GHz and optical frequency regions, the measured optical refractive index data were analyzed using the CM equation [16]. The dielectric constant (ε_opt_) at the optical frequencies is determined using the molar polarizability of electrons associated with constituent molecules in the glasses. On the other hand, the dielectric constant (ε_GHz_) at the GHz frequencies is determined using the total polarizability, including contributions from the electronic (α_e_) and ionic (α_i_) molar polarizabilities in the glasses [16]. Therefore, the ε_opt_ and ε_GHz_, are related to the α_e_, α_i_, and total polarizability, α_D_ = α_e_ + α_i_, as shown in the following equations:

(11) $$\frac{\left(\varepsilon_{\mathrm{opt}}-1\right)}{\left(\varepsilon_{\mathrm{opt}}+2\right)}=\frac{4\pi }{{3V}_{m}}N_{A}\alpha_{e}$$

(12) $$\frac{\left(\varepsilon_{\mathrm{GHz}}-1\right)}{\left(\varepsilon_{\mathrm{GHz}}+2\right)}=\frac{4\pi }{{3V}_{m}}N_{A}\left(\alpha_{e}+\alpha_{i}\right)$$

**Figure 9:**
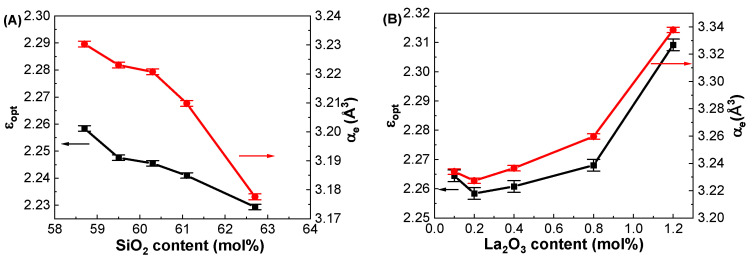
Variations in optical dielectric constants (ε_opt_) and electronic polarizabilities (α_e_) with respect to the SiO_2_ and La_2_O_3_ contents in the (**A**) GDMC-Si and (**B**) GDMC-La glass systems.

As noted before, the dielectric constants (ε_GHz_) of the glasses were measured at 1 GHz using the parallel plate method. The ε_opt_ of the glasses was calculated using the equation ε_opt_ = n_opt_^2^ from the optical refractive indices measured at 633 nm by considering a negligibly smaller extinction coefficient (K = αλ/4π, where α is the absorption coefficient and λ is the wavelength of the light) compared to the refractive index in the visible region. The calculated ε_opt_ values are shown in Table 4. Figure 9A shows the ε_opt_ of the GDMC-Si glasses for different SiO_2_ contents, and it decreased from 2.258 to 2.229 with increasing the substitution of SiO_2_ for Al_2_O_3_ in the glasses. Figure 9B shows the ε_opt_ of the GDMC-La glasses for different concentrations of La_2_O_3_. The ε_opt_ was found to increase from 2.264 to 2.309 with the substitution of La_2_O_3_ for (Al_2_O_3_ + MgO) in the glasses. The α_e_ of the present glasses was calculated from the measured refractive index data and molar volume using Equations (1) and (11) and are tabulated in Table 4. The cation ion polarizabilities (in Å^3^), Si^4+^: 0.033, Al^3+^: 0.054, B^3+^: 0.002, Ca^2+^: 0.469, Mg^2+^: 0.094, and La^3+^: 1.32, were used for the calculation [8]. As seen from Figure 9A,B, the α_e_ decreased from 3.230 to 3.178 Å^3^ and increased from 3.234 to 3.338 Å^3^ with the increase in SiO_2_ from 58.7 to 62.7 mol% and La_2_O_3_ from 0.1 to 1.2 mol%, respectively. On the other hand, the α_i_ of the present glasses was obtained using Equations (11) and (12) by subtracting α_e_ from α_D_. The calculated α_i_ values for both glass systems are shown in Table 4. The α_i_ values decreased from 2.980 to 2.892 Å^3^ and increased from 2.756 to 2.892 Å^3^ with the increases in SiO_2_ from 58.7 to 62.7 mol% and La_2_O_3_ content from 0.1 to 1.2 mol% in the glasses, respectively. The decrease in α_i_ in the GDMC-Si glasses with the substitution of SiO_2_ for Al_2_O_3_ is caused by the decrease in the ionic [AlO_4_]^−^ tetrahedra that are replaced by the covalent [SiO_4_] tetrahedra [41]. In GDMC-La glass series, the increase in α_i_ is due to the relative increase in polarizable NBOs caused by the incorporation of La_2_O_3_ [30,31,32]. Notably, the α_i_ values of the GDMC-La glasses were found to be lower in comparison with those of the reference glass (GDMC-Si59). This implies that the degree of ionic bonding can be reduced with the incorporation of La_2_O_3_ species in the alkali-free aluminoborosilicate glasses. The oxygen ion electronic polarizability (α_O2-_(n_o_)) and optical basicity (Λ (n_o_)) of the glasses were calculated using Equations (5) and (8) by subtracting the cation polarizabilities from α_e_. The average α_O2-_(n_o_) and Λ (n_o_) values for the GDMC-Si and GDMC-La glass systems were found to be 1.444 and 0.514 Å^3^, respectively. Interestingly, the α_e_ values obtained for the present glasses are close to those of the commercial fused silica (2.95 Å^3^) [16] and Pyrex (3.02 Å^3^) [16], indicating the suitably of the present glasses for use as low-dielectric material in the application of PCBs.

**Figure 10:**
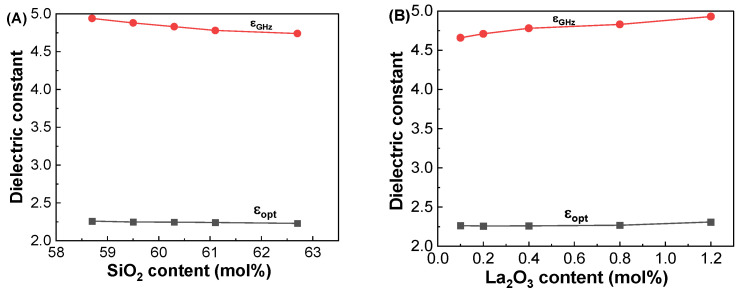

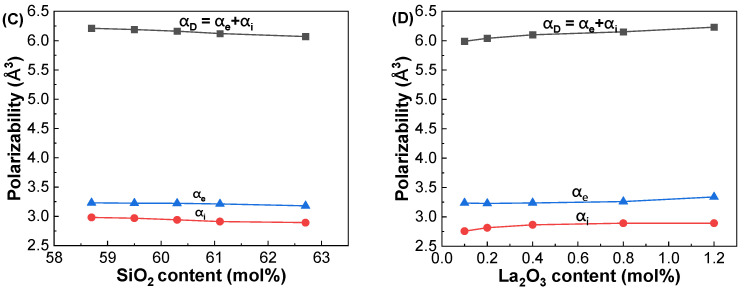
Variations in dielectric constants and polarizabilities at 1 GHz and at 633 nm with respect to the SiO_2_ and La_2_O_3_ contents in the (**A**,**C**) GDMC-Si and (**B**,**D**) GDMC-La glass systems.

Figure 10A,B depict the dielectric constants at 1 GHz and at 633 nm with respect to the SiO_2_ and La_2_O_3_ contents in the glasses, respectively. The polarizabilities calculated from the ε_GHz_ and ε_opt_ are plotted with respect to the SiO_2_ and La_2_O_3_ contents in the studied glasses, as shown in Figure 10C,D. It is worthy to note that the ε_GHz_ values were found to be much larger than those of the ε_opt_ in two series of glasses. This is because the ionic polarizability (α_i_) contributes additionally to the ε_GHz_ at GHz frequencies in the glasses as shown in Figure 10C,D. This indicates that higher dielectric constants at GHz frequencies can be expected in glasses with more ionic components.

## 4. Conclusions

We analyzed the dielectric and polarizability properties of the alkali-free aluminoborosilicate glasses with respect to the SiO_2_ and La_2_O_3_ contents in the two different frequencies at 1 GHz and at 633 nm. It was found that the ε_GHz_ decreased and increased with the increases in SiO_2_ and La_2_O_3_ contents in the GDMC-Si and GDMC-La glass systems, respectively. A good interrelationship was observed between the ε_GHz_ and the parameters α_D_, α_O2−_, and Λ in both the glass systems. Both of the present series of glasses exhibited a trend of ε_GHz_ increasing with increases in α_D_ and α_O2−_. The α_O2−_ parameter is regarded to govern the overall dielectric properties of glasses. The α_O2−_ decreased from 2.392 to 2.382 Å^3^ and increased from 2.301 to 2.347 Å^3^ with increasing SiO_2_ and La_2_O_3_ contents in the glasses. The dielectric constants (ε_opt_) and electronic polarizabilities (α_e_) at the optical frequencies were estimated for two series of glasses, and the contributions of the electronic (α_e_) and ionic (α_i_) polarizabilities to the dielectric constants were analyzed. The α_i_ dominated the dielectric response in the glasses at GHz frequencies. The decreasing ε_GHz_ in the glasses was confirmed by the decreasing α_i_. The GDMC-La glasses showed a lower α_i_ values than that of the reference GDMC-Si59 glass. The results show that the dielectric property is impacted by the glass composition. The present analysis would be useful for understanding the mechanisms contributing to the dielectric properties of glasses at GHz frequencies and for designing new glass compositions in high-speed communication applications. Due to their low dielectric property, they can also be useful for various applications in the GHz frequency range as dielectric filters, resonators, and antennas.

## Figures and Tables

**Table 1:** Chemical compositions of the alkali-free aluminoborosilicate glasses.

Glass Label	Glass Composition (mol%)					
	SiO_2_	Al_2_O_3_	B_2_O_3_	CaO	MgO	La_2_O_3_
GDMC-Si58	58.7	10.90	19.7	5.5	5.20	0
GDMC-Si59	59.5	10.20	19.6	5.5	5.20	0
GDMC-Si60	60.3	9.50	19.5	5.5	5.20	0
GDMC-Si61	61.1	8.80	19.4	5.5	5.20	0
GDMC-Si62	62.7	7.40	19.2	5.5	5.20	0
GDMC-La0.1	59.5	10.13	19.6	5.5	5.17	0.1
GDMC-La0.2	59.5	10.07	19.6	5.5	5.13	0.2
GDMC-La0.4	59.5	9.93	19.6	5.5	5.07	0.4
GDMC-La0.8	59.5	9.67	19.6	5.5	4.93	0.8
GDMC-La1.2	59.5	9.40	19.6	5.5	4.80	1.2

**Table 2:** Physical properties of the GDMC-Si and GDMC-La glass systems.

Glass Label	Physical Property			
	ρ (g/cm^3^)	V_m_ (cm^3^/mol)	ρ_ox_ (mol/cm^3^)	V_ox_ (cm^3^/mol)
GDMC-Si58	2.366 ± 0.003	27.589 ± 0.006	0.0797 ± 0.0001	12.546 ± 0.002
GDMC-Si59	2.346 ± 0.003	27.695 ± 0.006	0.0791 ± 0.0001	12.640 ± 0.002
GDMC-Si60	2.334 ± 0.003	27.708 ± 0.006	0.0788 ± 0.0001	12.693 ± 0.002
GDMC-Si61	2.325 ± 0.003	27.685 ± 0.006	0.0786 ± 0.0001	12.729 ± 0.002
GDMC-Si62	2.311 ± 0.003	27.591 ± 0.006	0.0782 ± 0.0001	12.779 ± 0.002
GDMC-La0.1	2.369 ± 0.003	27.529 ± 0.009	0.0796 ± 0.0001	12.561 ± 0.001
GDMC-La0.2	2.375 ± 0.003	27.564 ± 0.009	0.0795 ± 0.0001	12.572 ± 0.001
GDMC-La0.4	2.389 ± 0.003	27.605 ± 0.009	0.0794 ± 0.0001	12.584 ± 0.001
GDMC-La0.8	2.417 ± 0.003	27.691 ± 0.009	0.0793 ± 0.0001	12.608 ± 0.001
GDMC-La1.2	2.449 ± 0.003	27.728 ± 0.009	0.0793 ± 0.0001	12.609 ± 0.001

**Table 3:** Dielectric constants (ε_GHz_) and polarizability properties of the GDMC-Si and GDMC-La glass systems at the frequency of 1 GHz.

Glass Label	ε_GHz_ at 1 GHz	α_D_ (Å^3^)	α_O2−_ (Å^3^)	Λ
GDMC-Si58	4.94 ± 0.02	6.21 ± 0.01	2.392 ± 0.003	0.972 ± 0.001
GDMC-Si59	4.88 ± 0.02	6.19 ± 0.01	2.395 ± 0.003	0.973 ± 0.001
GDMC-Si60	4.83 ± 0.02	6.16 ± 0.01	2.391 ± 0.003	0.972 ± 0.001
GDMC-Si61	4.78 ± 0.02	6.12 ± 0.01	2.383 ± 0.003	0.969 ± 0.001
GDMC-Si62	4.74 ± 0.02	6.07 ± 0.01	2.382 ± 0.003	0.969 ± 0.001
GDMC-La0.1	4.66 ± 0.02	5.99 ± 0.01	2.301 ± 0.005	0.944 ± 0.002
GDMC-La0.2	4.71 ± 0.02	6.04 ± 0.01	2.315 ± 0.005	0.949 ± 0.002
GDMC-La0.4	4.78 ± 0.02	6.10 ± 0.01	2.332 ± 0.005	0.954 ± 0.002
GDMC-La0.8	4.83 ± 0.02	6.15 ± 0.01	2.334 ± 0.005	0.955 ± 0.002
GDMC-La1.2	4.93 ± 0.02	6.23 ± 0.01	2.347 ± 0.005	0.959 ± 0.002

**Table 4:** Optical refractive indices (n_o_), optical dielectric constants (ε_opt_), and electronic polarizabilities (α_e_) of the GDMC-Si and GDMC-La glass systems.

Glass Label	Refractive Index, n_o_				ε_opt_	α_e_ (Å^3^)	α_i_ (Å^3^)
	633 nm	830 nm	1310 nm	1550 nm			
GDMC-Si58	1.5028 ± 0.0005	1.4978 ± 0.0005	1.4900 ± 0.0005	1.4875 ± 0.0005	2.258 ± 0.001	3.230 ± 0.001	2.980 ± 0.001
GDMC-Si59	1.4992 ± 0.0003	1.4947 ± 0.0003	1.4867 ± 0.0003	1.4841 ± 0.0003	2.248 ± 0.001	3.223 ± 0.001	2.967 ± 0.001
GDMC-Si60	1.4985 ± 0.0002	1.4939 ± 0.0002	1.4861 ± 0.0002	1.4836 ± 0.0002	2.246 ± 0.001	3.221 ± 0.001	2.939 ± 0.001
GDMC-Si61	1.4970 ± 0.0003	1.4929 ± 0.0003	1.485 ± 0.0003	1.4825 ± 0.0003	2.241 ± 0.001	3.210 ± 0.001	2.910 ± 0.001
GDMC-Si62	1.4931 ± 0.0005	1.4886 ± 0.0005	1.4808 ± 0.0005	1.4782 ± 0.0005	2.229 ± 0.001	3.178 ± 0.001	2.892 ± 0.001
GDMC-La0.1	1.5048 ± 0.0002	1.4999 ± 0.0002	1.492 ± 0.0002	1.4894 ± 0.0002	2.264 ± 0.002	3.234 ± 0.002	2.756 ± 0.002
GDMC-La0.2	1.5028 ± 0.0001	1.498 ± 0.0001	1.4902 ± 0.0001	1.4877 ± 0.0001	2.258 ± 0.002	3.227 ± 0.002	2.813 ± 0.002
GDMC-La0.4	1.5036 ± 0.0001	1.4989 ± 0.0001	1.4911 ± 0.0001	1.4886 ± 0.0001	2.261 ± 0.002	3.236 ± 0.002	2.863 ± 0.002
GDMC-La0.8	1.5060 ± 0.0003	1.5013 ± 0.0003	1.4935 ± 0.0003	1.491 ± 0.0003	2.268 ± 0.002	3.260 ± 0.002	2.890 ± 0.002
GDMC-La1.2	1.5196 ± 0.0002	1.5147 ± 0.0002	1.5063 ± 0.0002	1.5038 ± 0.0002	2.309 ± 0.002	3.338 ± 0.002	2.892 ± 0.002

## Data Availability

Data are contained within the article.
